# Reliable Web-Based Auditory Cognitive Testing: Observational Study

**DOI:** 10.2196/58444

**Published:** 2024-12-09

**Authors:** Meher Lad, John-Paul Taylor, Timothy David Griffiths

**Affiliations:** 1 Translational and Clinical Research Institute Newcastle University Newcastle upon Tyne United Kingdom; 2 NIHR Newcastle Biomedical Research Centre Newcastle University Newcastle upon Tyne United Kingdom; 3 Biosciences Institute Newcastle University Newcastle upon Tyne United Kingdom; 4 Wellcome Centre for Human Neuroimaging University College London London United Kingdom

**Keywords:** auditory testing, hearing loss, cognitive testing, auditory, observational study, older adult, hearing, questionnaire, auditory cognitive testing, in person, web-based setting, auditory memory, Pearson, female, women, audiology

## Abstract

**Background:**

Web-based experimentation, accelerated by the COVID-19 pandemic, has enabled large-scale participant recruitment and data collection. Auditory testing on the web has shown promise but faces challenges such as uncontrolled environments and verifying headphone use. Prior studies have successfully replicated auditory experiments but often involved younger participants, limiting the generalizability to older adults with varying hearing abilities. This study explores the feasibility of conducting reliable auditory cognitive testing using a web-based platform, especially among older adults.

**Objective:**

This study aims to determine whether demographic factors such as age and hearing status influence participation in web-based auditory cognitive experiments and to assess the reproducibility of auditory cognitive measures—specifically speech-in-noise perception and auditory memory (AuM)—between in-person and web-based settings. Additionally, this study aims to examine the relationship between musical sophistication, measured by the Goldsmiths Musical Sophistication Index (GMSI), and auditory cognitive measures across different testing environments.

**Methods:**

A total of 153 participants aged 50 to 86 years were recruited from local registries and memory clinics; 58 of these returned for web-based, follow-up assessments. An additional 89 participants from the PREVENT cohort were included in the web-based study, forming a combined sample. Participants completed speech-in-noise perception tasks (Digits-in-Noise and Speech-in-Babble), AuM tests for frequency and amplitude modulation rate, and the GMSI questionnaire. In-person testing was conducted in a soundproof room with standardized equipment, while web-based tests required participants to use headphones in a quiet room via a web-based app. The reproducibility of auditory measures was evaluated using Pearson and intraclass correlation coefficients, and statistical analyses assessed relationships between variables across settings.

**Results:**

Older participants and those with severe hearing loss were underrepresented in the web-based follow-up. The GMSI questionnaire demonstrated the highest reproducibility (*r*=0.82), while auditory cognitive tasks showed moderate reproducibility (Digits-in-Noise and Speech-in-Babble *r*=0.55 AuM tests for frequency *r*=0.75 and amplitude modulation rate *r*=0.44). There were no significant differences in the correlation between age and auditory measures across in-person and web-based settings (all *P*>.05). The study replicated previously reported associations between AuM and GMSI scores, as well as sentence-in-noise perception, indicating consistency across testing environments.

**Conclusions:**

Web-based auditory cognitive testing is feasible and yields results comparable to in-person testing, especially for questionnaire-based measures like the GMSI. While auditory tasks demonstrated moderate reproducibility, the consistent replication of key associations suggests that web-based testing is a viable alternative for auditory cognition research. However, the underrepresentation of older adults and those with severe hearing loss highlights a need to address barriers to web-based participation. Future work should explore methods to enhance inclusivity, such as remote guided testing, and address factors like digital literacy and equipment standards to improve the representativeness and quality of web-based auditory research.

## Introduction

Web-based experimentation has allowed researchers to reach a large number of participants in a short time. This has increased the statistical power of studies and the reliability of their findings. Web-based recruitment platforms like Amazon Mechanical Turk and Prolific.co have become common venues for data collection, and stimulus creation and presentation are simplified through mediums like Gorilla, PsychoPy, and Qualtrics [1,2]. This has resulted in a shift in research culture, where an increasing number of laboratories are trying to answer their research questions by using these resources. The COVID-19 pandemic accelerated this process [3].

Web-based auditory testing has also increased during this time, and a range of studies have evidenced the reliability and replicability of this method [4,5]. Auditory experiments rely on well-controlled delivery of sounds with headphone usage, as the variability of background noise during the sound presentation, equipment, and choice of device used for presenting sounds can dramatically impact the user experience. For example, in a web-based setting, it is not easy to verify whether a participant is using headphones, which attenuate background noise and ensure accurate delivery of sound requiring 2 audio channels, without visual verification. This introduces concerns regarding privacy. Many tests have been devised to overcome this problem [6]. This has led to a number of successful replications of auditory perceptual experiments [5,7]. However, a number of these studies have been conducted on young, motivated individuals who have volunteered for such experiments. People with severe hearing loss who may depend on hearing aids may not be able to physically accommodate headphones easily, as listening without hearing aids may need to compensate for individual hearing impairments [8].

Some researchers have expressed concerns about the population being sampled from web-based databases [9]. Participants tend to be less representative of local populations. In-person research has a lack of diversity in participation where people with limited literacy skills are often excluded from health research, and this may be exacerbated when digital literacy is taken into consideration [10]. Older adults may be further marginalized, although it has been shown that web-based cognitive testing has been successfully implemented with adults older than 65 years of age [11]. Nevertheless, the effectiveness of web-based testing on older adults with hearing difficulty is unclear.

There is a paucity of auditory cognitive research, using stimuli assessing speech-in-noise (SIN) hearing, for example, in older populations with a range of hearing abilities. Previous work has used verbal stimuli, which are easier to perceive than simple or complex auditory stimuli below the level of speech [12]. We have learned that participants are able to complete demanding tasks on a web-based platform with a particular experimental setup [13]. Learning effects of complex sound stimuli have also been studied in young and older adults recruited from Prolific.co successfully [14]. A combination of such stimuli with verbal stimuli has been studied in a similar participant group [15]. Whether reliable, web-based, auditory cognitive testing can be performed, using similar stimuli, in a “real-world” population has not been previously attempted to our knowledge.

This first part of this exploratory study aimed to assess if there were population characteristics, such as age and hearing status, that were more likely to lead to web-based experimentation. We then assessed the reproducibility of auditory cognitive metrics in the participants who participated in person and on the web. We asked participants to complete 2 verbal SIN perception tasks, for digits and sentences, and 2 auditory memory (AuM) tasks, for different sound features. We compared whether participants scored similarly on a self-reported questionnaire about musical sophistication, the Goldsmiths Musical Sophistication Index (GMSI), to scores obtained using computerized behavioral experiments for auditory cognition such as SIN measures and AuM [16]. We hypothesized better reproducibility of the questionnaire compared to the auditory cognitive tasks. We also assessed if there were differences in correlation coefficients between auditory measures and those that were shared across the 2 task settings, such as age and hearing thresholds.

For the second part of the study, we tested relationships between the auditory cognitive metrics that were identified previously by the in-person experimentation and were reproducible in a web-based group of participants. This included the participants from the first part and adults from the PREVENT study, a cohort of participants who were recruited to study risk factors for dementia [17]. We wished to examine whether web-based auditory cognitive experiments would reproduce findings that we had found between the GMSI and AuM for frequency precision and between SIN thresholds for sentence-in-babble perception and AuM [18,19]. During these in-person experiments, stricter auditory experimental procedures were used, and we expected participants to be more consistent, in this context, across in-person and web-based experiments compared to the web-based computerized tests.

## Methods

### Participants

A total of 153 older adults were recruited (from 300 invitations, equaling a response rate of 51%) from a local Newcastle University volunteer database, the Join Dementia Research registry, and from friends and family of people attending memory clinics for a dementia workup. Phone calls and emails were the methods of invitation. Inclusion criteria included the absence of a neurological or psychiatric condition at the time of participation and age of 50 years or older. Lack of fluency in English was the only exclusion criterion.

The age range of participants was 50-86 years with a mean of 67 (SD 10) years. A total of 23 participants were active hearing aid users. All participants agreed to take part in a web-based, follow-up assessment between 3 and 6 months after their initial in-person assessment. Participants who conducted the web-based assessments did not report any subjective change in their hearing abilities or any new medical conditions affecting their cognition. In total, 58 (37.9% of the 153 in-person attendees) of these participants took part in a web-based repeat assessment. The performance of these participants was used to determine the reproducibility of auditory cognitive tests in person versus in a web-based setting. We aimed to recruit as many participants as possible, and therefore, no sample size calculation was performed.

A total of 89 additional participants (from 500 invitations, equaling a response rate of 18%) were invited from another participant cohort, the PREVENT study. This study was originally developed to establish midlife risk factors for dementia in a multicenter UK cohort of participants. The aim of the web-based study was to test whether previously published findings could be replicated on the web. These were studies showing an association between AuM and sentence-in-noise perception ability and between AuM for frequency precision and musical sophistication [17-19]. The Newcastle and the PREVENT cohort were combined to provide a dataset to analyze web-based auditory cognitive metrics. Participants were compensated with US $15 shopping vouchers for in-person testing and US $7 vouchers for web-based experimentation.

**Figure 1:**
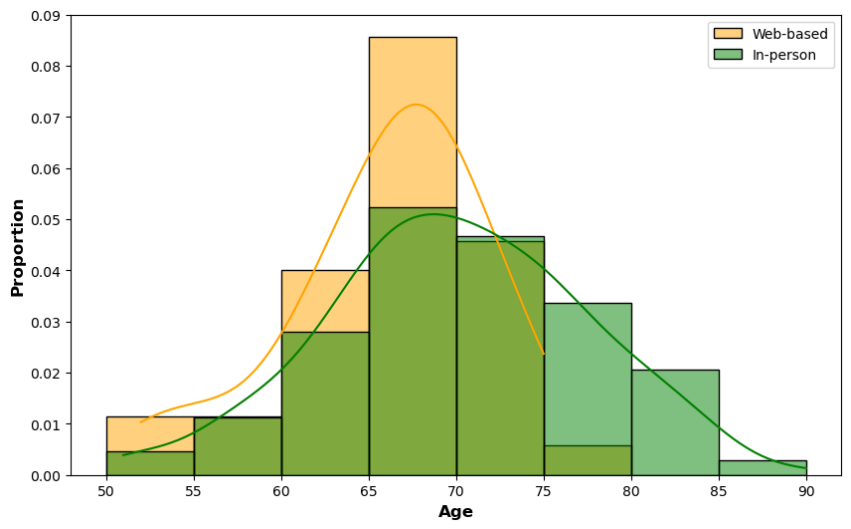
Age in the in-person (n=153; green) versus web-based (n=58; yellow) cohort of participants. People older than 70 years of age were less likely to participate in web-based auditory cognitive experimentation.

The mean age of participants in this group was 65 (SD 8) years. Of the 153 participants, 56.8% (n=87) were women, 5.2% (n=8) of participants left school at the age of 15 years, 69.9% (n=107) completed school and had a university degree, and 24.2% (n=37) had multiple degrees. In total, 11.1% (n=17) had normal hearing, 18.9% (n=87) had mild hearing loss, 54.2% (n=83) had moderate hearing loss, and 15.7% (n=24) had severe hearing loss. As shown in Figure 1, there were no participants older than 75 years of age who participated on the web. Only 4 (11%) out of 37 hearing aid users took part in the web-based experimentation session. Due to the small number of participants present in this group, further subgroup analysis was not conducted. In the study of web-based participants, across the Newcastle and PREVENT cohorts, the average age was 59 (SD 7.7) years. The sample included 75 female and 47 male participants. As these participants did not have pure-tone audiometry performed, we were unable to grade the severity of hearing loss like for the in-person participants.

### Stimuli and Equipment

In-person testing was conducted in a soundproof room using a Dell desktop computer with Sennheiser HD 201 circumaural headphones. An external sound card was used to process and deliver auditory stimuli, and acoustic stimuli were presented at 70 dBA. All auditory cognitive tasks and the GMSI questionnaire, both of which are described in detail in the next section, were coded in Javascript and displayed with a Google Chrome web browser. The web page was designed as a fully automated single-page application, where all stimulus generation and user interaction were determined by processes that occurred “client-side.” The web page was hosted by Google Cloud Platform using Firebase Hosting. A Firebase Realtime Database was used to store anonymized results that were linked to each participant ID, which they used to access the testing portal at home at the follow-up assessment. At home, participants were instructed to use a desktop, laptop, or tablet device with headphones and perform the test in a quiet room with no distractions. Each participant was sent a link that directed them to the web-based testing platform. Hearing aid users were instructed to use over-ear headphones that do not interfere with the aids, if available, or to increase the computer device’s sound to a comfortable level before beginning the auditory tests.

### Experimental Procedure

Each participant had a 1-hour visit to the Auditory Laboratory at the Newcastle University Medical School. Pure tone audiometry (PTA) testing was performed on both ears from 250 Hz to 8 kHz at octave intervals for air conduction using an Interacoustics AS608e screening audiometer. Tones were manually presented as short bursts twice starting at 30 dB hearing level (HL) then increased in 5 dB increments until comfortably audible if necessary. Then 5 dB HL reductions were made until the tone was not audible. This process was repeated twice, and the lowest audible volume was chosen as the value for a particular frequency. If maximum amplification at 100 dB HL could not be perceived then this was used as the ceiling value at a particular frequency. The overall mean of high-frequency values between 4 and 8 kHz for the best ear was taken as the threshold value for an individual for further analysis. This value was chosen as high-frequency thresholds are suspected to deteriorate first in age-related hearing loss and previous research from our group has suggested that PTA thresholds in this range correlate with SIN difficulties [20,21]. Hearing status was determined as normal if the mean threshold was below 20 dB HL, mild if between 20 and 40 dB HL, moderate if 40-60 dB HL hearing loss, and severe if >80 dB HL.

The Digits-in-Noise (DIN) task involved participants listening to 3 digits on a background of speech-shaped white noise, created using white noise using the long-term average speech spectrum of speech stimuli between 80 and 10,000 Hz. Participants had 2 practice trials at the beginning of the task to familiarize themselves with the stimuli at a signal-to-noise ratio (SNR) of 10 dB. An adaptive 1-up, 1-down psychophysical paradigm was implemented, whereby a correct response resulted in the SNR being reduced and an incorrect one caused the SNR to increase. The starting SNR was 0 dB and the step sizes decreased from 5 to 2 dB after 3 reversals, which then reduced to 0.5 dB after 3 more reversals. The run terminated after 10 reversals and the SNR at the last 5 reversals was averaged to calculate the DIN threshold for each participant. Lower SNR values indicated a better performance. As with the DIN task, participants had 2 practice trials at the beginning of the task to familiarize themselves with the stimuli at an SNR of 10 dB. The Speech-in-Babble (SIB) task consisted of participants listening to sentences on a background of 16-talker babble as described previously by our research group [18,21]. Target sentences had the form <name> <verb> <number> <adjective> <noun> (eg, “Alan gives four pretty flowers”) and participants had to click on the correct word from a list of 5 columns (10 options for each word) shown on the screen with the same structure. The SIB threshold was determined using the same adaptive threshold procedure used in the DIN test. The starting parameters and adaptive design were exactly the same as the DIN task.

**Figure 2:**
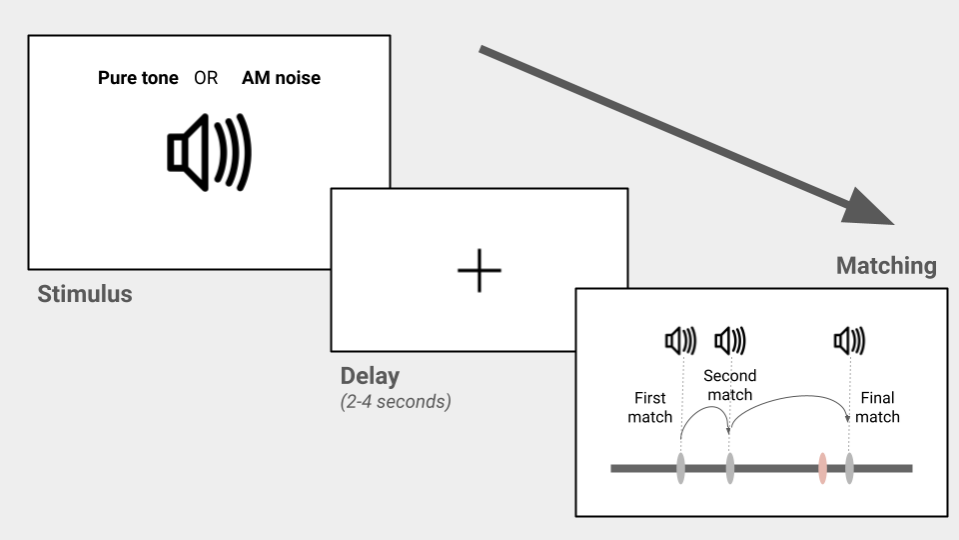
Auditory memory experiment. An auditory (pure tone or amplitude-modulated noise) stimulus is presented for 1 second; then, after a delay of 2 to 4 seconds, participants can match sounds using a horizontal scale on the screen. The scale is linked to the parameter of interest (frequency for pure tone or AM rate) that can generate the original stimulus after exploring the parameter space to “find” the stimulus. The figure shows an auditory matching trial where the participant’s “final match” (rightmost dark gray marker on the scale) is shown in comparison to where the original stimulus (orange marker on the scale) is actually located. In this example, the participant first clicked on the scale to make a “first match” (which produced a sound linked to the parameter at that location), then a “second match,” and then a “final match.” The discrepancy between the “final match” location parameter and that of the original stimulus gives an “error” for each trial that can be used to calculate the auditory working memory “precision,” the inverse of the SD of errors from a trial target, for all auditory trials. AM: amplitude modulation.

AuM was tested using nonspeech stimuli as previously described (Figure 2; Lad et al [19]). A 1-second tone or amplitude-modulated white noise stimulus was presented to a participant, after which they were asked to “find” the sound on a horizontal scale on a computer screen. Participants had to move a mouse and click on the line to produce a sound at that location. They could make as many clicks as they wanted with no set time limit. After they were satisfied with their choice, they would advance to the next trial by pressing the “Enter” key on a keyboard. Frequencies that determined the pure-tone sounds were chosen from a uniform distribution between 440 and 880 Hz, and amplitude modulation (AM) rates for the white noise stimulus were 5-20 Hz with a sinusoidal function used to apply this modulation. This parameter space, with an addition of 10% at either end of the scale, for each sound feature was mapped to the pixels of the horizontal scale on the screen during the matching phase. Hanning windows were applied to all synthetic sounds to avoid clicks and the beginning and end of the stimuli. The task consisted of 32 trials with the frequency and AM rate matching trials being interleaved. Participants had a short break after 16 trials. A Gaussian function was used to estimate the SD of the errors in each trial across the whole experiment and the inverse of this value, the precision, was used for further analysis. Thus, one obtains a precision for frequency AuM (“AuM (F)”) and AM rate AuM (“AuM (A)”). Studies in vision have found that this measure better reflects the memory resource a participant can allocate in a given task [22]. Participants had 2 practice trials with each stimulus—2 for AuM (F) and 2 for AuM (A)—at the beginning of the task to familiarize themselves with the stimuli.

Finally, participants completed the short version of the GMSI questionnaire (Multimedia Appendix 1) consisting of 38 questions on paper as a test of musicality [16]. The GMSI is a self-report inventory that assesses individual differences in musical sophistication. It is said to measure the ability to engage with music in a comprehensive manner that does not just include musical instrumentation or training. For the purposes of this study, the GMSI was used as it has been previously used alongside the auditory cognitive measures in this study in an in-person setting [19]. We have previously shown a significant relationship between GMSI scores and AuM for frequency precision. This includes the total score of 266—the sum of the scores of the 5 domains of “Active Engagement,” “Perceptual Abilities,” “Musical Training,” “Singing Abilities,” and “Emotions.” This questionnaire was used as a control of internal consistency between in-person and web-based experimentation, as we expected the change in testing modality to least affect a participant’s ability to answer multiple-choice questions about their musical behaviors compared to the auditory tests.

### Statistical Analysis

AuM scores were log-transformed to achieve normal distributions. All other variables were normally distributed and converted to *z* scores for further analysis. Any missing values were not imputed.

The chi-square tests were used to calculate the difference in proportions in age groups and hearing groups between in-person and web-based participants. Pearson correlation coefficients were then used to measure the strength and direction of the linear relationship between auditory metrics. To evaluate the reproducibility of web-based versus in-person testing across different age groups, we used correlation coefficients and intraclass correlation coefficients. Steiger test was used to assess whether the correlation coefficients between in-person and web-based auditory metrics with shared variables such as age and PTA thresholds were significantly different.

The relationship between 2 variables while controlling for the effect of another variable (eg, age) was performed after using linear regression to account for the influence of the control variable. This was used to assess the relationship between auditory cognitive metrics in person and then via a web-based platform. Finally, differences in correlation coefficients between these 2 sets of variables were calculated using bootstrapping. This was performed using repeated resampling of the data with replacement 1000 times. This allowed for the calculation of the mean difference and 95% CIs for the correlation differences. Significant differences between correlation coefficients were established if the CIs did not overlap. Due to the exploratory nature of this study, statistical correction for multiple corrections was not applied. All analyses were carried out using the Python (Python Software Foundation) programming language using Jupyter notebooks. *SciPy* and *Pingouin* packages were used.

### Ethical Considerations

The Oxford C NHS Research Ethics Committee (21/SC/0139) approved this study on June 12, 2021. All participants gave written consent to participate and publish data and for secondary analysis without additional consent. All study data are anonymous. In-person participants obtained US $15 vouchers and web-based participants received US $7 vouchers for participation. This study was not registered as it was not a randomized controlled trial.

## Results

### In-Person Versus Web-Based Auditory Cognitive Testing

Figure 2 shows the normalized age distribution of the in-person participant group and those who returned for web-based testing. The proportion of participants with different degrees of hearing loss is also shown. There was a significant difference in the proportion of participants older than 70 years of age between the web-based and in-person groups, (*χ*^2^_1_=9.40; *P*=.002), with a higher proportion of older participants in the in-person group. For the web-based participants, the proportion of those with moderate hearing loss was 54.1% (n=83), while the proportion with severe hearing loss was 16.2% (n=25). The mean hearing loss level was 45.84 (SD 18.84) dB HL for web-based participants and 52.32 (SD 23.03) dB HL for in-person participants. There was a statistically significant difference between the proportions of moderate and severe hearing loss categories among web-based participants (*χ*^2^_1_=6.02; *P*=.01). For the in-person participants, the proportion of those with moderate hearing loss was 33.5% (n=51), and the proportion with severe hearing loss was 35.4% (n=54). There was no statistically significant difference in the proportions of participants with moderate and severe hearing loss among in-person participants (*χ*^2^_1_=1.07; *P*=.30).

**Figure 3:**
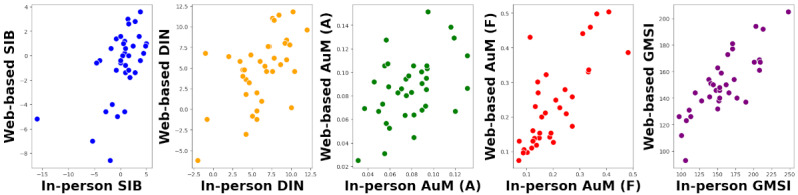
Participant (n=58) data for various auditory cognitive measures and the GMSI questionnaire in in-person and web-based settings. In-person data are shown on the x-axis, whereas web-based metrics are shown on the y-axis. AuM (A): auditory memory precision for amplitude modulation rate; AuM (F): auditory memory precision for frequency; DIN: digits-in-noise task threshold; GMSI: Goldsmiths Musical Sophistication Index score; SIB: Speech-in-Babble threshold.

Table 1 shows the mean and SD values of the auditory cognitive tests in both settings. There were no statistical differences between the mean values between in-person and web-based auditory measures. We assessed the reproducibility of various auditory and cognitive measures conducted in both laboratory and web-based settings using the Pearson correlation coefficient and intraclass correlation coefficients. The results indicated moderate reproducibility for DIN (*r*=0.55, 95% CI 0.27-0.74; *P*<.001) and SIB (*r*=0.55, 95% CI 0.27-0.74; *P*<.001). AuM (F) demonstrated good reliability (*r*=0.75, 95% CI 0.56-0.87; *P*<.001), while AuM (A) showed lower reliability (*r*=0.44, 95% CI 0.14-0.67; *P*=.007). Notably, the GMSI exhibited the highest test-retest reliability among the measures (*r*=0.82, 95% CI 0.67-0.90; *P*<.001). The reproducibility of the GMSI, with a correlation coefficient (*r*) of 0.82, was significantly greater than that of the SIB (mean difference=0.25, 95% CI 0.05-0.46; *P*<.05) and AuM (A) (mean difference=0.37, 95% CI 0.08-0.69; *P*<.05). The correlation coefficient of AuM (F) was not significantly higher than AuM (A) (mean difference=0.31, 95% CI –0.02 to 0.67; *P*=.07) and the DIN was not significantly more reproducible than SIB (mean difference=–0.25, 95% CI –0.36 to 0.29; *P*=.81). The visualizations for these relationships are shown in Figure 3.

**Table 1:** In-person and web-based auditory cognitive performance.

	Participants (n=58)		Correlation coefficient	Intraclass correlation coefficient
	In person, mean (SD)	Web based, mean (SD)		
GMSI^a^ (total score)	156 (36)	151 (23)	0.82	0.72
DIN^b^ (dB)^c^	6.35 (2.8)	5.42 (3.2)	0.55	0.49
SIB^d^ (dB)	2.45 (2.8)	2.01 (2.1)	0.55	0.62
AuM (F)^e^ (a.u)^f^	1.52 (0.6)	1.34 (0.7)	0.75	0.67
AuM (A)^g^ (a.u)	2.52 (0.4)	2.42 (0.4)	0.44	0.43

^a^GMSI: Goldsmiths Musical Sophistication Index.

^b^DIN: Digits-in-Noise task threshold.

^c^dB: decibels.

^d^SIB: Speech-in-Babble threshold.

^e^AuM (F): auditory memory precision for frequency.

^f^a.u: arbitrary units.

^g^AuM (A): auditory memory precision for amplitude modulation rate.

We tested whether there were differences in correlation coefficients between age, PTA thresholds measured in person, and auditory cognitive metrics measured in both settings using the Steiger test. There were no significant differences between the correlations between age and any auditory metric in the web-based and in-person settings. Specifically, these included the DIN (t_57_=0.165; *P*=.87), SIB (t_57_=0.162; *P*=.87), AuM (A) (t_57_=–0.170; *P*=.87), and AuM (F) (t_57_=0.073; *P*=.94). Additionally, there were no significant differences between the correlations between PTA thresholds and any auditory metrics across settings. These included the DIN (t_57_=0.005; *P*=.99), SIB (t_57_=0.031; *P*=.98), AuM (A) (t_57_=–0.012; *P*=.99), and AuM (F) (t_57_=0.154; *P*=.88).

### Web-Based Auditory Cognitive Testing Associations

We tested whether auditory cognitive associations between variables obtained in person were reproducible on the web with a larger group of web-based participants. The latter included the addition of a new group of participants, from the PREVENT study, who had not previously performed the task. This was done to improve the statistical power of the web-based findings. We examined the reproducibility of the association between AuM for frequency and GMSI scores and between SIB and AuM when conducted in person and web-based. This has been studied previously by our group on an in-person participant group [18,19].

**Figure 4:**
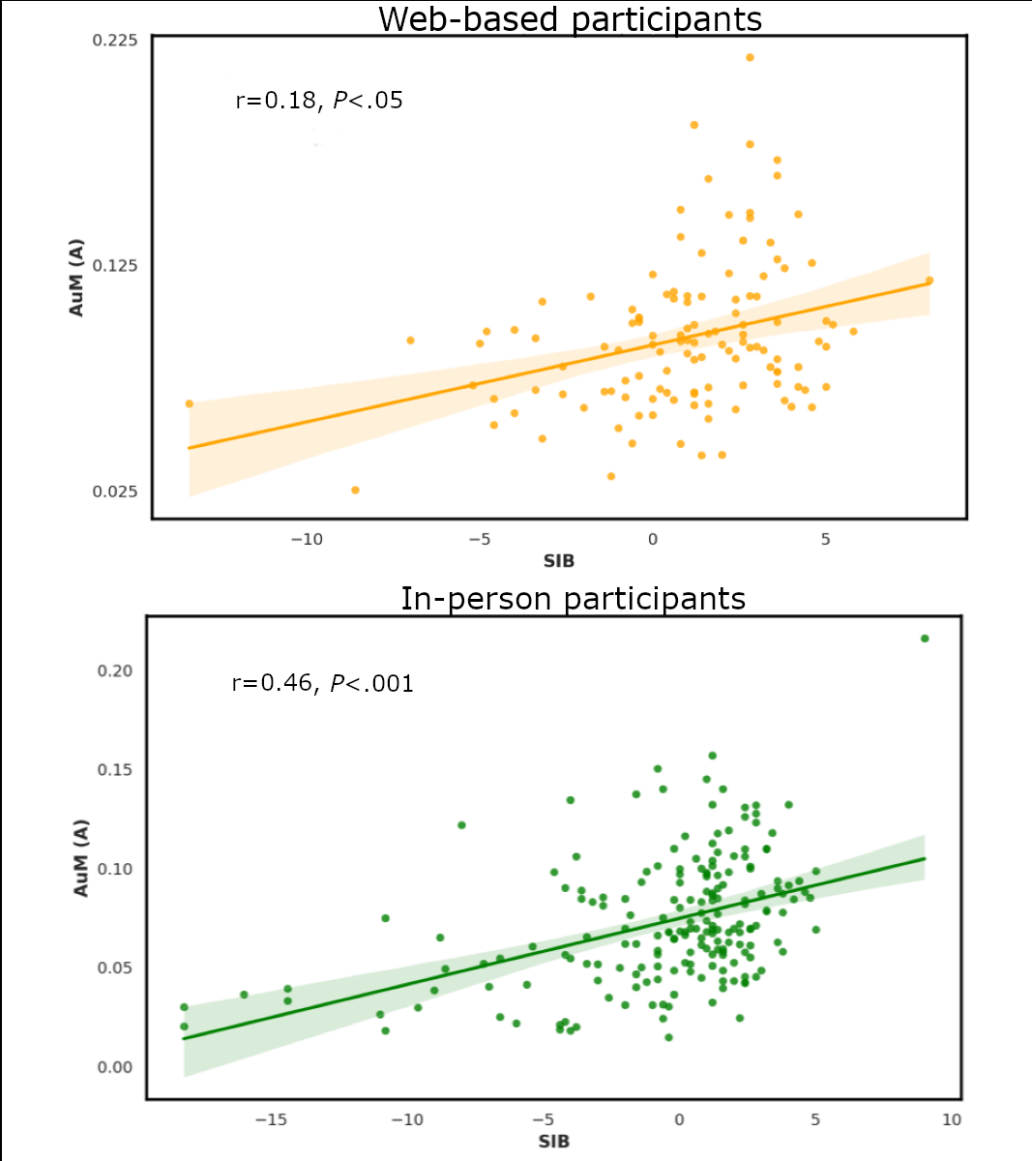
The relationship between SIB perception ability and AuM (A) in web-based (n=147; top panel: orange) and in-person (n=153; bottom panel: green) participants. Shaded areas indicate CIs for the regression lines of best fit. There is greater variability for web-based participant data compared to in-person data. AuM (A): auditory memory precision for amplitude modulation; SIB: Speech-in-Babble threshold.

The correlation coefficients for the relationship between SIB and AuM (A) were statistically significant for in-person testing (*r*=0.46, 95% CI 0.34-0.56; *P*<.001) and web-based testing (*r*=0.18, 95% CI 0.0-0.34; *P*<.05; Figure 4). Bootstrapping was used to generate 95% CIs for the correlation coefficients to test if they were significantly different at the significance level with an α of .05. We did not find any differences across the 2 settings for this relationship. Similarly, the correlation coefficients for the relationship between GMSI scores and AuM (F) were statistically significant for in-person testing (*r*=0.52, 95% CI 0.42-0.61; *P*<.001) and web-based testing (*r*=0.47, 95% CI 0.32-0.6; *P*<.001). We did not find any differences across the 2 settings for this relationship. This suggested that there were no differences in the correlation of these auditory measures in in-person or web-based settings.

## Discussion

### Principal Findings

The principal findings of this study were that remote auditory testing of cognition is reliable when conducted on the web. However, web-based participation may not be fully representative of the participants who may take part in person. We found that older participants and those with severe hearing loss were less likely to participate on a web-based platform. Furthermore, we found that the questionnaire-based test, the GMSI, had the best reproducibility as compared to the auditory cognitive tasks, which did not differ significantly when conducted in person or on the web. Importantly, previously published findings were reproducible on the web suggesting that web-based testing may be a suitable avenue to test auditory cognition.

### Comparison to Prior Work

Although this study has not been conducted previously, our experience with web-based auditory experimentation was similar to previous work in the field [5,14,15]. Older adults in the “real-world” were able to successfully negotiate a self-directed, web-based auditory experimentation portal and give reliable results in comparison to in-person testing. Our study differed from the previous work in the type and range of stimuli across the same experimental paradigm. For example, we tested the perception of digits and sentences on a noisy background and sounds manipulated by frequency and temporal fluctuations. This allowed us to test different types of auditory stimuli across the same type of experiment (SIN perception and AuM). We did not find any differences in the reproducibility of any of these measures across in-person or web-based settings. Importantly, there was no significant difference when comparing the relationships of these metrics with shared variables across both settings such as age and hearing thresholds.

The focus of this study was to engage participants with a range of hearing abilities that are recruited for in-person studies. Despite an increase in web-based research methods, there may be an underrepresentation of participants who lack adequate digital literacy or find it difficult to interact with computer-generated stimuli. In particular, people with hearing loss may rely more on visual cues to understand information, which may be absent from typical auditory research performed on healthy young adults without hearing difficulty [23]. Although we did not explore the qualitative reasons behind the reduced proportion of participants with severe hearing loss in our web-based cohort, these participants were also more likely to be hearing aid users, which could have interfered with their use of headphones. Data on whether participants were using Bluetooth-enabled devices that allowed connection to their electronic devices were not collected. Additionally, to maximize participation in the study, we gave volunteers up to 6 months to complete their web-based experiment. Although this may have improved participant retention to a degree, we did not remeasure their PTA thresholds to ensure the peripheral hearing was the same as in the in-person experiment. This may have improved the reproducibility of the web-based auditory cognitive metrics.

We also found no differences in the reproducibility of auditory cognitive stimuli with particular sound parameters. There could have been better reproducibility for AuM (F) due to the invariance of sounds with a pitch to changes in timbre over different electronic devices [24]. The pitch of a sound is also less likely to be affected if it is played on headphones or speakers and due to the sound cards in electronic devices. For example, the frequency space from which the pure tones were generated was from 440 to 880 Hz, which is captured by most audio devices. Speech-based sounds and amplitude-modulated white noise stimuli would have only been limited by the bandwidth of the electronic devices and equipment. This could have affected the performance in the SIN tasks and AuM (A) task on the web and contributed to the lower correlation coefficient. However, these were not statistically different. Further work is needed to clarify the reasons for this.

The observed similarities between amplitude-modulated noise stimuli and speech in conversations highlight their importance in understanding auditory processing, particularly in relation to SIN perception ability. Both modalities exhibit temporal amplitude fluctuations critical for investigating auditory system functions without background noise [25]. Modulated noise mimics speech’s natural rhythms, aiding in the study of temporal processing in speech. Cognitive engagement is essential in both, requiring attention and working memory to decode or track fluctuations; however, the presence of linguistic and phonetic information in speech is not found in the generated sounds of the AuM task. Despite these differences, the correlation between nonspeech stimuli in auditory modulation tasks and verbal SIN tasks indicates underlying auditory cognitive abilities shared across both domains, bridging the gap between speech and nonspeech auditory processing. This suggests a broader scope of auditory cognition beyond specific linguistic content, highlighting fundamental auditory processing mechanisms applicable across different auditory stimuli. The reproducibility of this relationship across the in-person and web-based settings suggests that these fundamental connections between the stimuli can be investigated on the web.

### Strengths and Limitations

The home environment of a participant may affect a participant’s performance in a number of ways. Although there were instructions to set the volume level to one that was comfortable in our task, participants may alter this through the course of the tasks. The home environment is also prone to background fluctuations in noise that may not be observed in strict laboratory conditions. These interactions may affect the listening ability of participants despite the sounds being at a comfortable level [26]. Finally, despite using headphones, the sound quality that may have been transmitted through them could have varied between participants resulting in any changes in sound quality mentioned. It was impossible to monitor this accurately during this study. Despite all of these challenges, it is promising for the auditory measures used in this study to display reproducibility across a range of different SIN stimuli and AuM tasks with different sound features. However, the participants in this study were recruited from a local university volunteer database, which may introduce a selection bias as these individuals are more likely to have prior experience with research participation. Therefore, while our findings provide valuable insights, they may not be fully generalizable to the broader population and this limitation should be considered when interpreting the results.

The strengths of this study include the inclusion of “real-world” older adult participants in independent cohorts, with a range of hearing abilities, to perform in-person and web-based auditory experiments. This allowed us to accurately assess the differences in within-subject parameters that are attributable to web-based testing. We also used multiple types of verbal and nonverbal stimuli to test auditory cognitive abilities, which allowed us to find differences in performance for particular sound features, like frequency versus amplitude modulation, in both settings.

### Future Work

Future work could include an assessment of the reasons for which participants did not participate in the web-based session despite agreeing to participate in this initially. There may have been other factors, rather than those related to hearing, that influenced these decisions for each participant. Other improvements to the study design include remote guided testing, where the participant performs the task on their home computer but through a screen share [27]. This would ensure that the home environment and equipment could “pass” a minimum standard to perform the tasks. Recruitment of a larger group of participants who return for web-based testing may allow further analysis to assess the reproducibility of the auditory measures across different age groups and hearing loss severity levels. This will allow us to assess if these are factors that affect the reproducibility of certain results.

## Appendix

Multimedia Appendix 1 Goldsmiths Musical Sophistication Index (GMSI) questionnaire.

## Abbreviations

AM: amplitude modulation
AuM: auditory memory
AuM (A): auditory memory for amplitude modulation rate
AuM (F): auditory memory for frequency
DIN: Digits-in-Noise
GMSI: Goldsmiths Musical Sophistication Index
HL: hearing level
PTA: pure tone audiometry
SIB: Speech-in-Babble
SIN: speech-in-noise
SNR: signal-to-noise ratio

## Data Availability

The datasets generated and analyzed during this study are available from the corresponding author on reasonable request.
