# Supplementary material for: Reliable Web-Based Auditory Cognitive Testing: Observational Study
Source: J Med Internet Res. 2024 Dec 9;26:e58444. doi: 10.2196/58444 (PMC11667740; doi:10.2196/58444)
Supplement: Multimedia Appendix 1 [file jmir_v26i1e58444_app1.pdf]

The Goldsmiths Musical Sophistication Index, v1.0

October 11, 2012

**Please circle the most appropriate category:**	**1 Completely Disagree**	**2 Strongly Disagree**	**3 Disagree**	**4 Neither Agree nor Disagree**	**5 Agree**	**6 Strongly Agree**	**7 Completely Agree**
1. I spend a lot of my free time doing music-related activities.	1	2	3	4	5	6	7
2. I sometimes choose music that can trigger shivers down my spine.	1	2	3	4	5	6	7
3. I enjoy writing about music, for example on blogs and forums.	1	2	3	4	5	6	7
4. If somebody starts singing a song I don't know, I can usually join in.	1	2	3	4	5	6	7
5. I am able to judge whether someone is a good singer or not.	1	2	3	4	5	6	7
6. I usually know when I'm hearing a song for the first time.	1	2	3	4	5	6	7
7. I can sing or play music from memory.	1	2	3	4	5	6	7
8. I'm intrigued by musical styles I'm not familiar with and want to find out more.	1	2	3	4	5	6	7
9. Pieces of music rarely evoke emotions for me.	1	2	3	4	5	6	7
10. I am able to hit the right notes when I sing along with a recording.	1	2	3	4	5	6	7

**Please circle the most appropriate category:**	**1 Completely Disagree**	**2 Strongly Disagree**	**3 Disagree**	**4 Neither Agree nor Disagree**	**5 Agree**	**6 Strongly Agree**	**7 Completely Agree**
11. I find it difficult to spot mistakes in a performance of a song even if I know the tune.	1	2	3	4	5	6	7
12. I can compare and discuss differences between two performances or versions of the same piece of music.	1	2	3	4	5	6	7
13. I have trouble recognizing a familiar song when played in a different way or by a different performer.	1	2	3	4	5	6	7
14. I have never been complimented for my talents as a musical performer.	1	2	3	4	5	6	7
15. I often read or search the internet for things related to music.	1	2	3	4	5	6	7
16. I often pick certain music to motivate or excite me.	1	2	3	4	5	6	7
17. I am not able to sing in harmony when somebody is singing a familiar tune.	1	2	3	4	5	6	7
18. I can tell when people sing or play out of time with the beat.	1	2	3	4	5	6	7
19. I am able to identify what is special about a given musical piece.	1	2	3	4	5	6	7
20. I am able to talk about the emotions that a piece of music evokes for me.	1	2	3	4	5	6	7

**Please circle the most appropriate category:**	**1 Completely Disagree**	**2 Strongly Disagree**	**3 Disagree**	**4 Neither Agree nor Disagree**	**5 Agree**	**6 Strongly Agree**	**7 Completely Agree**
21. I don't spend much of my disposable income on music.	1	2	3	4	5	6	7
22. I can tell when people sing or play out of tune.	1	2	3	4	5	6	7
23. When I sing, I have no idea whether I'm in tune or not.	1	2	3	4	5	6	7
24. Music is kind of an addiction for me - I couldn't live without it.	1	2	3	4	5	6	7
25. I don't like singing in public because I'm afraid that I would sing wrong notes.	1	2	3	4	5	6	7
26. When I hear a piece of music I can usually identify its genre.	1	2	3	4	5	6	7
27. I would not consider myself a musician.	1	2	3	4	5	6	7
28. I keep track of new music that I come across (e.g. new artists or recordings).	1	2	3	4	5	6	7
29. After hearing a new song two or three times, I can usually sing it by myself.	1	2	3	4	5	6	7
30. I only need to hear a new tune once and I can sing it back hours later.	1	2	3	4	5	6	7
31. Music can evoke my memories of past people and places.	1	2	3	4	5	6	7

Please circle the most appropriate category:

32. I engaged in regular, daily practice of a musical instrument (including voice) for **0 / 1 / 2 / 3 / 4-5 / 6-9 / 10 or more** years.

33. At the peak of my interest, I practiced **0 / 0.5 / 1 / 1.5 / 2 / 3-4 / 5 or more** hours per day on my primary instrument.

34. I have attended **0 / 1 / 2 / 3 / 4-6 / 7-10 / 11 or more** live music events as an audience member in the past twelve months.

35. I have had formal training in music theory for **0 / 0.5 / 1 / 2 / 3 / 4-6 / 7 or more** years.

36. I have had **0 / 0.5 / 1 / 2 / 3-5 / 6-9 / 10 or more** years of formal training on a musical instrument (including voice) during my lifetime.

37. I can play **0 / 1 / 2 / 3 / 4 / 5 / 6 or more** musical instruments.

38. I listen attentively to music for 0-15 min / 15-30 min / 30-60 min / 60-90 min / 2 hrs / 2-3 hrs / 4 hrs or more per day.

39. The instrument I play best (including voice) is ____

Please tick one of the following:

Occupational status

- ☐ Still at School
- ☐ At University
- ☐ In Full-time employment
- ☐ In Part-time employment
- ☐ Self-employed
- ☐ Homemaker/full time parent
- ☐ Unemployed
- ☐ Retired

What is the musical genre you mainly listen to?
(tick only one box)

- ☐ Rock/Pop
- ☐ Jazz
- ☐ Classical Music

What is the Highest educational qualification you have attained?

☐ Did not complete any school qualification

☐ Completed first school qualification at about 16 years (e.g. GCSE/Junior High School)

☐ Completed Second qualification (e.g A levels/ High School)

☐ Undergraduate degree or professional qualification

☐ Postgraduate degree

☐ I am still in education

If you are still in education, what is the highest qualification you expect to obtain?

☐ First school qualification (e.g. GCSE / Junior High School)

☐ Post-16 vocational course

☐ Second school qualification (e.g. A-levels / High School)

☐ Undergraduate degree or professional qualification

☐ Postgraduate degree

☐ Not applicable

Anonymous ID:____________
(please write down the last three letters of your surname, the day you were born (2 digits) and todays day (8 digits). Example: Adam Smith, born 13.02.1982, taken the test 05.03.2011 and Anonymous ID: ith1305032011)

Your age : ______ years.

Gender: **Female / Male**

Nationality:

Country in which you spent the formative years of your childhood and youth:

Country of current residency:

Email address (permanent email address, optional): ______________________________________

Name (optional): ______________________________________

☐ Please tick the box only if you dont want to be contacted about this project again in the future.
